# Pro‐inflammatory signalling and gut‐liver axis in non‐alcoholic and alcoholic steatohepatitis: Differences and similarities along the path

**DOI:** 10.1111/jcmm.15182

**Published:** 2020-04-21

**Authors:** Trenton Glaser, Leonardo Baiocchi, Tianhao Zhou, Heather Francis, Ilaria Lenci, Giuseppe Grassi, Lindsey Kennedy, Suthat Liangpunsakul, Shannon Glaser, Gianfranco Alpini, Fanyin Meng

**Affiliations:** ^1^ Texas A&M University College of Medicine College Station TX USA; ^2^ Liver Unit Department of Medicine University of Rome Tor Vergata Rome Italy; ^3^ Department of Medical Physiology Texas A&M University College of Medicine Bryan TX USA; ^4^ Richard L. Roudebush VA Medical Center Indianapolis IN USA; ^5^ Division of Gastroenterology and Hepatology Department of Medicine Indiana University School of Medicine Indianapolis IN USA

**Keywords:** ASH, gut‐liver axis, inflammation, microRNA, NASH, steatohepatitis

## Abstract

Non‐alcoholic fatty liver disease (NAFLD) and alcohol‐associated liver disease (ALD) represent a spectrum of injury, ranging from simple steatosis to steatohepatitis and cirrhosis. In humans, in fact, fatty changes in the liver, possibly leading to end‐stage disease, were observed after chronic alcohol intake or in conditions of metabolic impairment. In this article, we examined the features and the pro‐inflammatory pathways leading to non‐alcoholic and alcoholic steatohepatitis. The involvement of several events (hits) and multiple inter‐related pathways in the pathogenesis of these diseases suggest that a single therapeutic agent is unlikely to be an effective treatment strategy. Hence, a combination treatment towards multiple pro‐inflammatory targets would eventually be required. Gut‐liver crosstalk is involved not only in the impairment of lipid and glucose homoeostasis leading to steatogenesis, but also in the initiation of inflammation and fibrogenesis in both NAFLD and ALD. Modulation of the gut‐liver axis has been suggested as a possible therapeutic approach since gut‐derived components are likely to be involved in both the onset and the progression of liver damage. This review summarizes the translational mechanisms underlying pro‐inflammatory signalling and gut‐liver axis in non‐alcoholic and alcoholic steatohepatitis. With a multitude of people being affected by liver diseases, identification of possible treatments and the elucidation of pathogenic mechanisms are elements of paramount importance.

## 1 INTRODUCTION

**Figure 1:**
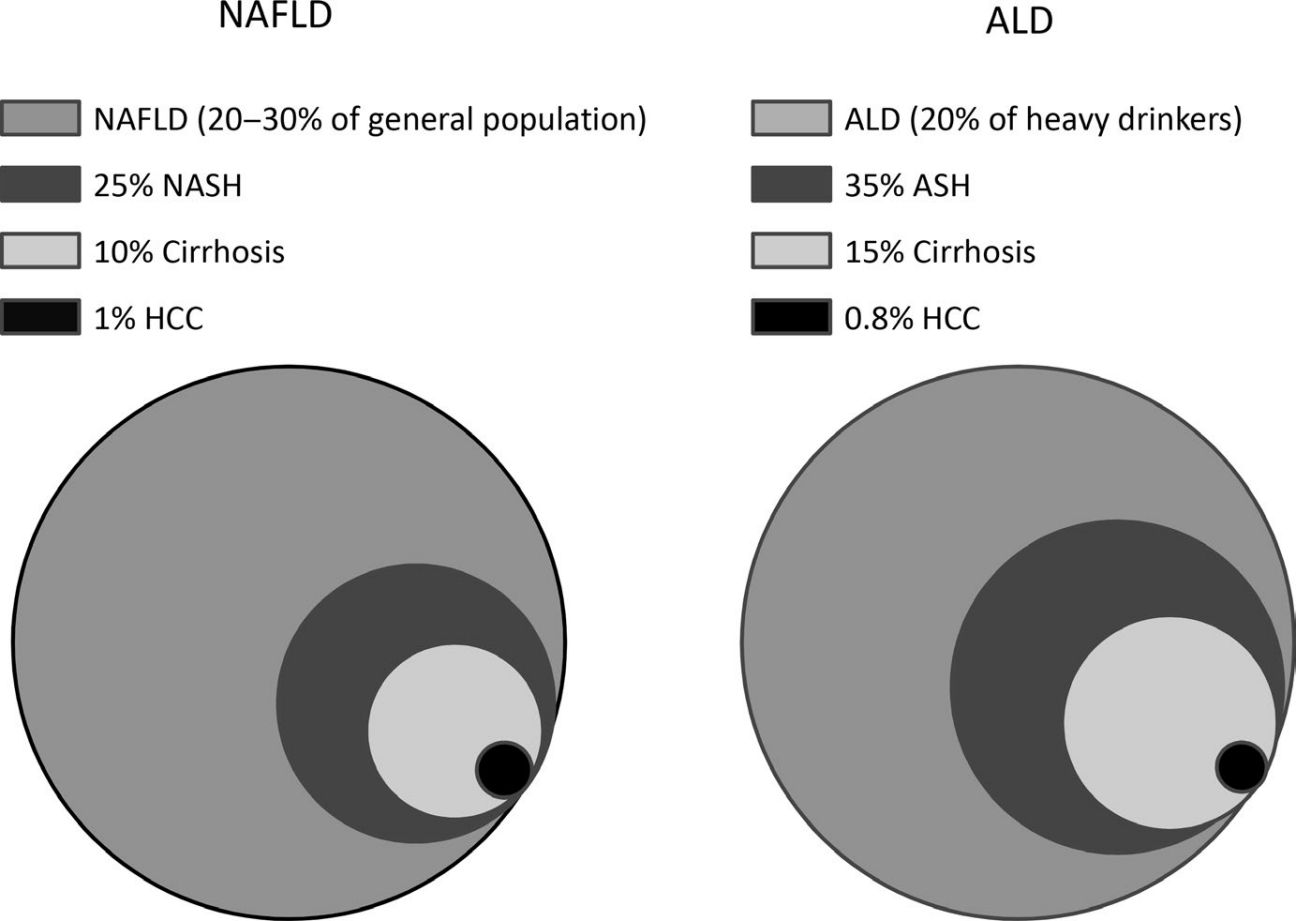
Spectrum of liver diseases associated with NAFLD or ALD with the corresponding prevalence. Different pathologic affections encountered in NAFLD and ALD are reported in figure for comparison. Circle area is proportional to the prevalence of any specific condition. In the legend, prevalence is reported in percentage with the corresponding disease. Data were obtained from references.^6^, ^7^ ALD, alcoholic liver disease; ASH, alcoholic steatohepatitis; HCC, hepatocellular carcinoma; NAFLD, non‐alcoholic fatty liver disease; NASH, non‐alcoholic steatohepatitis

In the United States, nearly 35% of the population is obese [body mass index (BMI) >30] and another 6.3% is considered to have morbid obesity (BMI > 40 or more); the majority of these people have or may develop insulin resistance and/or metabolic syndrome, both of which are related to non‐alcoholic fatty liver disease (NAFLD).^1^, ^2^ NAFLD affects 1%‐20% of the US population, whereas non‐alcoholic steatohepatitis (NASH) affects 1.1%‐14% of US population.^3^, ^4^ NAFLD itself is not necessarily harmful; however, it can lead to NASH that is characterized by prolonged chronic liver inflammation and injury. NASH is one of the leading causes of liver cirrhosis in the United States together with alcoholic liver disease (ALD). Regarding the latter, nearly 50% of all cirrhosis related deaths are associated with chronic alcohol consumption.^5^ Other types of ALD include fatty liver, alcoholic steatohepatitis (ASH) and cirrhosis.^6^ Approximately, 20%‐30% of subjects with chronic alcohol abuse may develop fatty liver, whereas 15% of patients may develop cirrhosis with 1% prevalence of hepatocellular carcinoma (HCC).^7^ Type of liver affections associated with NAFLD or ALD with the corresponding prevalence are reported in Figure 1. The pathogenesis of NASH and ALD appears similar from a clinical point of view, since both diseases are characterized by fatty infiltration of the liver. In this context, clinical phenotypes suggest similarities including (a) different expression of disease in a single patient, (b) similar clinical presentation and (c) occurrence of multiple factors (“hits”) for progression to end‐stage liver disease.

There is a translational link between NAFLD and ALD. NAFLD is a liver disease which includes fatty liver (NAFL) which is considered benign disease, steatohepatitis (NASH), which causes inflammation and accumulation of fat and scar tissue in the liver and can progress to advanced fibrosis, cirrhosis and its complication like HCC.^8^ Although a similar condition can occur in people who abuse alcohol, NASH usually occurs in those who drink little to no alcohol. ALD spectrum comprises simple steatosis, alcoholic hepatitis, and cirrhosis and HCC. There is significant overlap of the translational link between NAFLD and ALD, and clinical presentation depends upon the stage of liver disease. NAFLD patients are mostly asymptomatic and diagnosed to have fatty liver while undergoing routine health examination.^8^ ALD requires significant history of alcohol intake which is supportive by serum and biochemical tests. In both NAFLD and ALD patients, liver enzymes are usually not significantly changed. Liver biopsy is required for diagnosis of NASH as it is a histological diagnosis and sometimes in alcoholic hepatitis for confirmation if diagnosis is uncertain. Some serum/ultrasound markers and prognostic scores have been developed for shunning liver biopsy in evaluation and treatment response of NASH and alcoholic hepatitis patients.^9^

In a study involving 52 patients (26 with NASH and 26 with ALD), few parameters discriminated NASH from ALD. The most obvious factors were the nutritional status and features of visceral fat deposition. Histological rates of fibrosis and necrosis in NASH and ALD were similar, whereas patients with NASH exhibited higher levels of steatosis. The degrees of lipid peroxidation were similar,^10^ despite the increased cholesterol serum levels in patients with NASH phenotypes. Although many obese people develop NASH, this disease has been also observed in non‐obese, lean subjects; on the other hand, only 35% of all heavy drinkers develop cirrhosis. These observations suggest that other factors may contribute to human NASH and ASH onset/progression (Table 1). In this context, possible contributing factors include the following: (a) lipotoxicity; (b) mitochondrial dysfunction and oxidative damage; (c) innate immune system; and (d) changes in the gut‐liver axis. Comparative pathogenesis of NASH and ASH as a function of different pathways involved and the role of the liver‐gut axis in these events will be outlined in this review.

**Table 1:** Comparison between NASH and ASH with regard to the mechanisms of liver injury

	NASH	ASH
Fatty liver determinants	↑ Lipolysis of adipose tissue^14^	↑ FAs synthesis ↓ FAs oxidation^17^, ^18^
Lipotoxicity	↑ Apoptosis for enhanced hepatic free FAs^22^ Formation of lipidic toxic intermediates^23^ FC‐induced liver damage^23^	Likely to occur but no studies exist
Determinants of mitochondrial dysfunction and oxidative stress	↓ Electronic transport chain activity and adenosine triphosphate (ATP) synthesis^29^ ROS and RNS‐induced degradation of mitochondrial factors^30^ Cardiolipin degradation with destabilization of mitochondrial enzymes^32^	NADH and acetaldehyde toxic damage CYP2E1‐induced ROS formation^17^, ^18^
Effects on innate immune system	Myd88‐dependent TLR‐4 activation^39^, ^42^ NLRP3 inflammasome assembly^42^	Myd88‐independent TLR‐4 activation^42^

Abbreviations: ASH, alcoholic steatohepatitis; FA, fatty acid; CYP2E1, cytochrome P450 2E1; FC, free cholesterol; Myd88, myeloid differentiation primary response 88; NADH, nicotinamide adenine dinucleotide; NASH, non‐alcoholic steatohepatitis; RNS, reactive nitrogen species; ROS, reactive oxygen species; TLR‐4, Toll‐like receptor 4.

## 2 FATTY LIVER

In normal conditions, even if the liver is involved in the management/disposal of an important traffic of lipids (mainly composed by triglycerides, and fatty acids), fat deposition in tissue does not occur. Low concentration of lipids in the graft is obtained by a fine balance between fat uptake/neo‐synthesis and fatty acid oxidation plus lipoprotein assembly/disposal.^11^ An imbalance in fat recruitment and its use in the liver determines an abnormal accumulation of lipids in hepatocytes thus resulting in fatty liver.^12^ The characteristic morphological feature (by histology or with imaging techniques) of this condition is represented by liver steatosis. Hepatic steatosis is found in patients with NAFLD, NASH and ALD and is diagnosed when more than 5% of liver cells contain fat at microscopic examination.^13^ However, there are several causes, with lower prevalence, that may determine fatty liver, including surgical, toxic, viral and inborn errors. In the course of steatosis, fat droplets are mainly composed by triglycerides for the enhanced accumulation and storage of fatty acids. The fatty acids pool can be increased by three main mechanisms: (a) lipolysis of visceral fat by intracellular lipases; (b) “de novo” liver lipid synthesis; and (c) dietary intake. While fatty liver is an essential marker of NAFLD and ALD, accumulation of lipids in hepatocytes is thought to occur with different mechanisms in these two conditions. In NAFLD patients, using a multiple stable‐isotope approach**,** it was shown that the majority of fat liver deposition (nearly 60%) is coming from increased lipolysis of adipose tissue, whereas dietary fats contribute for only 10%.^14^ In this setting, output of fat from adipose tissue has been demonstrated to be increased by the obese state and proportionate to insulin resistance.^15^ Even if increased lipolysis of adipose tissue has been suggested to be involved in ALD (in addition to NAFLD),^16^ during chronic ethanol consumption there is also a major contribution of alcohol oxidative processes in increasing nicotinamide adenine dinucleotide (NADH) that in turn stimulates increased fatty acid synthesis^17^ and inhibition of fatty acid oxidation.^18^ Whatever the specific molecular mechanisms of the onset are, fatty liver has been considered for both NAFLD and ALD the first step of a pathogenetic process possibly leading to liver inflammation/damage.

## 3 POSSIBLE FACTORS CONTRIBUTING TO PROGRESSION FROM FATTY LIVER TO NASH OR ASH

Progression from fatty liver to hepatic injury is coordinately regulated by a series of molecular events. In this perspective, the original “two hits” pathogenetic theory on the development of steatohepatitis^19^ has been proposed by the evidence that, more likely, several “parallel hits” contribute to liver damage evolution in NAFLD and ALD.^17^, ^20^ On the other hand, since steatohepatitis is a benign condition in a large number of patients, the possibility that NASH may be a different disease rather than a fatty liver evolution is not completely excluded. In the following sub‐paragraphs, the possible determinants of damage progression in NAFLD and ALD are described.

### 3.1 Lipotoxicity

With regard to fats, the increased injurious concentration of lipids and lipid derivatives in hepatic cells has been recognized to determine the so called “lipotoxicity”.^21^ In the case of NASH, increased liver input of free fatty acids (FFA) is able to determine hepatocyte apoptosis by both intrinsic and extrinsic pathways, evidenced by increased caspase and c‐Jun N‐terminal kinase (JNK) pathway activity.^22^ Also, the production of toxic intermediates (such as diacylglycerol, ceramide and sphingolipids) by dysregulated lipid metabolism further increases liver damage. With regard to lipids, another typical feature of NASH is represented by the accumulation of free cholesterol (FC) within the liver as observed in human tissue by lipidomic analysis.^23^ This event has been related to enhanced sterol regulatory element‐binding protein 2 (SREBP‐2) activity, likely triggered by endoplasmic reticulum (ER) stress.^24^ FXR agonists such as obeticholic acid have been shown to improve all parameters of early‐stage NASH, effects that were associated with increased LDL‐cholesterol.^25^ The mechanism by which FC accumulation may promote liver injury and inflammation is not completely clarified yet; however, stimulation of Kupffer cells, ER stress and mitochondrial dysfunction by FC has been observed in experimental models.^26^ While lipotoxicity is likely to occur in the course of ASH, this mechanism of injury was not extensively examined in this disease. Indirect evidence, coming from experimental studies, suggests that FA (in particular the unsaturated form) accumulation in the liver, by itself, may promote steatosis and inflammation.^27^ However, whether FA playing a role in inflammation processes during ALD remains to be assessed as suggested by a recent review.

### 3.2 Mitochondrial dysfunction and oxidative stress

Increased fatty content in the liver, during NAFLD, enhances oxidative compensatory events in the mitochondria.^28^ As the lipid accumulation exceeds the metabolic mitochondrial capacity, the electronic transport chain activity and adenosine triphosphate (ATP) synthesis are impaired, as demonstrated in rodents fed a choline‐deficient diet.^29^ This supports production of both reactive oxygen species (ROS) and reactive nitrogen species (RNS). These molecules further enhance mitochondrial injury as DNA, both mitochondrial and cellular, is damaged possibly affecting the synthesis of key mitochondrial factors such a peroxisome proliferator–activated receptor‐gamma coactivator (PGC)‐1alpha, mitochondrial transcription factor A (TFAM) and nuclear factor erythroid 2–related factor 2 (Nrf2).^30^ These findings identify that DNA in mitochondria is more prone to oxidative damage since this sub‐cellular structure does not hold histone or DNA regenerative properties. The progressive cascade of events finally determines a condition defined as mitochondrial dysfunction.^31^ Cardiolipin oxidation has also been suggested to have an important role in this process during NASH progression.^32^ In fact, a functional cardiolipin is thought to stabilize the structure and function of complex respiratory mitochondrial enzymes, being largely present (more than 20% of total lipids) in the inner mitochondrial membrane.^33^

However, this phospholipid given its chemical structure, characterized by several unsaturated groups, is highly prone to oxidative degradation possibly leading to important biochemical and morphological changes in mitochondria. Mitochondrial dysfunction, together with the consequent production of ROS and RNS, is considered as important steps for the progression of NAFLD, and pharmacological strategies targeting mitochondria are currently under examination.^34^ In the course of alcohol abuse, differently from NAFLD, oxidative injury and mitochondrial impairment of the liver are a direct consequence of ethanol metabolism. While alcohol dehydrogenase (ADH) determines the formation of NADH and acetaldehyde with toxic results, the major route leading to formation of ROS and oxidative damage is related to the induction of Cytochrome P450 2E1 (CYP2E1).^17^, ^18^ The latter enzyme is in fact increased up to 10‐fold in the liver of drinkers contributing to leakage of significant amounts of ROS during the conversion of ethanol in acetaldehyde. Finally, mitochondrial dysfunction develops in ASH since alteration of electron flow through the complexes of mitochondrial inner membrane is present, contributing to oxidative damage. Even if mitochondrial dysfunction is a common key element in the pathogenesis of ASH and NASH, morphological alterations of these organelles are visible only in NASH, suggesting a different, not yet elucidated, mechanism of mitochondrial damage in these two diseases.^35^

### 3.3 Innate immune system in the liver and inflammation

Several innate immune cells are present in the liver, including Kupffer cells (KCs), innate lymphoid cells (ILCs) and natural killer cells (NKs), which all contribute to the innate immune response of the graft. The liver innate immune system (IMS) has an important role in filtering and neutralizing exogenous deleterious agents, with KCs being the first line in detecting injurious components through pattern‐recognition receptors (PRRs), and then activating inflammatory response. However, IMS is also able to act as a promoter of inflammation in other stress (oxidative, metabolic) conditions. Among several PRRs, the Toll‐like receptor (TLR) family is certainly involved in NAFLD/NASH molecular processes. TLRs are able to react both to exogenous pathogen‐associated molecular patterns (PAMPs) and endogenous danger‐associated molecular patterns (DAMPs).^36^ Currently, 10 TLRs have been identified in humans.^37^ Among them, TLR4 seems to be the one mainly involved in NASH pathogenesis. Rodent models of TLR4 deficiency consistently demonstrated reduced NASH‐related liver injury^38^ and TLR4 or CD14 (a TLR4 co‐receptor) polymorphisms in human have been linked to NASH development and severity in some studies.^39^ Stimulation of TLR4 in the setting of NASH has been linked to NACHT, LRR and PYD domain‐containing protein 3 (NLRP3) inflammasome assembly and consequent onset and maintenance of inflammatory processes.^40^ In fact, NLRP3^−/−^ mice are protected by diet‐induced NASH, while human liver specimens of NASH subjects denoted increased gene expression of NLRP3 in comparison with those with steatosis only.^41^

**Figure 2:**
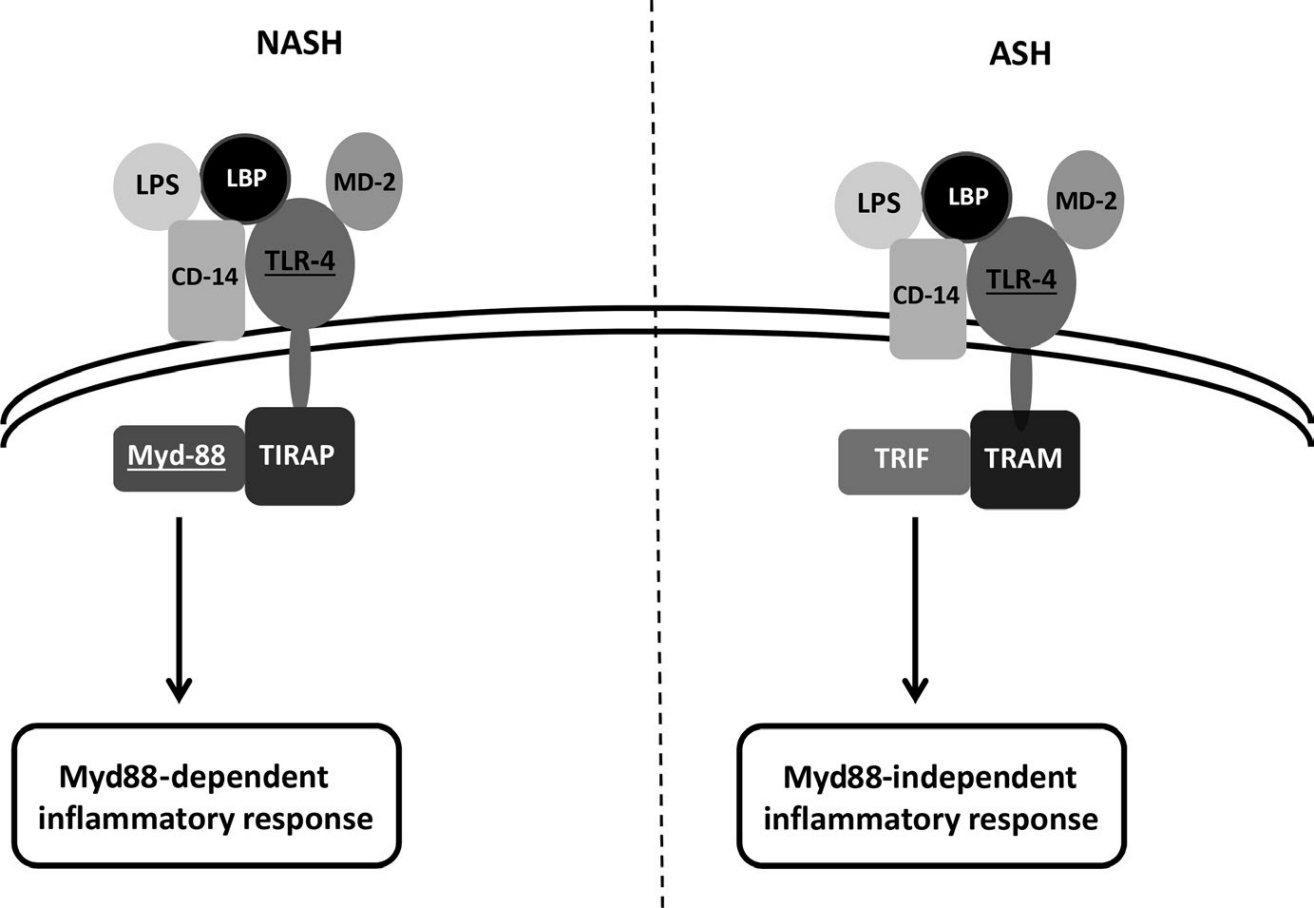
Schematic representation of TLR‐4 activation in the course of NASH or ASH. TLR‐4 activation, upon LPS stimulation, requires LBP and CD‐14 cooperation to facilitate LPS binding with TLR4/MD‐2 complex. The following intracellular processes are then different comparing NASH and ASH. In NASH, TLR4 oligomerizes and reacts with TIRAP and its protein adaptor Myd‐88 giving origin to a pro‐inflammatory cytokines response. On the other hand, during ASH, TLR4 reacts with TRAM coupling with its adaptor TRIF. Inflammatory response is then mainly represented by type 1 interferon, and interferon inducible genes. ASH, alcoholic steatohepatitis; LBP, LPS‐binding protein; LPS, lipopolysaccharide; Myd88, myeloid differentiation primary response 88; NASH, non‐alcoholic steatohepatitis; TLR4, Toll‐like receptor 4; TRAM, TRIF‐related adaptor molecule; TRIF, TIR domain‐containing adapter–inducing interferon‐β

TLR4 stimulation is thought to be of paramount importance also for ASH damage; however, transduction pathways after TLR4 stimulation are different between NASH and ASH. In the first condition, nuclear factor kappa‐light‐chain‐enhancer of activated B cells (NF‐κB) activation (and the consequent production of pro‐inflammatory cytokines) is obtained through the canonical myeloid differentiation primary response 88 (Myd88) pathway.^42^ On the other hand, in ASH inflammation is supported by a Myd88‐independent, TIR domain‐containing adapter–inducing interferon‐β (TRIF)‐related interferon regulatory factor 3 (IRF3) activation.^43^ A comparative representation of TLR4 Myd88‐dependent and Myd88‐independent pathways is depicted in Figure 2.

Another difference regards the possible NLRP3 inflammasome involvement in ASH injury as previously reported for NASH. Experimental data in fact observed (differently from NASH) decreased NLRP3 inflammasome activity in mouse macrophages and human mononuclear cell under ethanol treatment.^44^, ^45^ Ethanol‐induced inhibition of phosphorylation processes was recognized as a possible mechanism for inflammasome‐reduced activity, in this setting. However, the relationship between NLRP3 inflammasome and ethanol abuse remains controversial since in other organs, such as brain, a contribution of this pathway to the inflammatory process was reported.^46^

However, also considering the possible differences in the molecular pathogenesis of these diseases TLR‐4 activation seems to be of paramount importance in the onset of both NASH and ASH. Considering lipopolysaccharide (LPS), a component of Gram‐negative bacterial wall, is the main stimulator of TLR4, the possible role of gut‐liver axis and intestinal microbiota in NASH and ASH onset are extensively investigated at present.

## 4 GUT‐LIVER AXIS IN NASH AND ASH

### 4.1 Background

In recent years, our concept of gut‐liver axis has been extended, considering not only the canonical liver (bile‐mediated) effects on the gut absorptive process, but also those of gut products (mainly bacterial components and nutrients) reaching the liver through the portal vein. In fact, the liver is the first and more exposed organ to gut‐derived products. The gut contains 10‐100 trillion of microorganisms (10‐30 thousand different species) that compose the microbiota. The term microbiome instead defines the collective genomes and genetic products of microbiota.^47^ Since genetic composition of microbiome in humans should be relevant for the possible relationship with healthy or diseased conditions in the liver and other organs, the NIH Human Microbiome Project was launched in the past decade to characterized microbiome genetic.^48^

In normal conditions, with a functional intestinal barrier, even if portal vein determines a direct and rapid connection between gut and liver, intact bacteria are seldom observed in hepatic tissue.^49^ However, bacterial genetic products (mRNA) and LPS are easily detected in portal blood and liver, giving evidence of the possible influence of gut‐derived bacterial products on the liver activities in physiological conditions. Enhanced LPS levels in blood have been linked to obesity and insulin resistance through the so called “metabolic endotoxemia”.^50^

This may suggest dysbiosis as a possible contributing factor to fatty liver and later to NASH or ALD. A study was conducted to determine whether there was any relationship between hepatic lipid metabolism and microbiota composition. Rats were fed a high‐fat diet (HFD) while being treated with antibiotics (ab) to induce dysbiosis. Blood serum absorbance was measured in order to determine lipid concentrations. The HFD + ab fed group had significantly elevated levels of HDL, LDL, total cholesterol and triglyceride when compared with both the HFD and control groups, along with a significant shift in bacteria gut composition.^51^ This study shows that a change in microbiota composition increases lipid serum levels, which could lead to liver deposition of fats and evolution to steatohepatitis.

### 4.2 LPS receptors in NASH and ASH

Moreover, increased LPS levels were associated with fed or fasted conditions, while alterations of the gut barrier by increased assumption of ethanol or fats are related to enhanced translocation of microbe‐associated molecular patterns (MAMPs) to the liver.^52^ Liver interaction with microbial products would result in physiologic or pathologic effects according to different conditions.^53^ In this context, TLRs are of paramount importance with their reactivity with PAMPs as described above in regard to IMS. In fact, among PAMPs LPS is the most studied and main activator of TLR4.^54^ The latter has a major role for determining inflammatory response in the course of both NASH and ASH, and it has been considered an important biological sensor of circulating LPS since the past century.^55^ Stimulation of TLR‐4 receptor by LPS is a composite event involving the participation of other molecules including an LPS‐binding protein (LBP), CD14 and MD‐2. The final step consists in the interaction of the MD‐2/LPS complex with TLR‐4 receptor, determining its homo‐dimerization and activation of the signal cascade leading to inflammatory response through Myd88‐dependent or Myd88‐independent pathways.^56^ Taking into consideration these molecular pathways, the possible involvement of gut‐liver axis through the PAMPs/TLR‐4/pro‐inflammatory cytokines axis in the pathogenesis of inflammatory processes during NASH and ASH seems obvious. In addition, several evidence supports this view. First of all, increased circulating LPS levels were detected in both NASH and ASH.^57^, ^58^ This was associated with bacterial overgrowth or microbiota changes as well as increased intestinal permeability.^57^, ^58^ In human NASH, bowel injury has been described in both the intestinal epithelial barrier [(IEB) first‐line defence] and the gut vascular barrier [(GVB) second‐line defence].^59^ In this study, GVB damage was described as an early and important event in the onset of NASH, and pharmacological preservation of GVB integrity was proposed as a possible strategy for disease prevention. With regard to ASH, possible GVB injury was not examined and remains unknown.

A study was conducted in order to determine whether alcohol would affect the morphology of the duodenum. Data showed that with chronic ethanol consumption, the villi and brush border become significantly thinner than in control groups. Along with a shortening of the villi an almost 2× increase in mononuclear infiltrate cells is observed, suggesting an increased inflammatory response. Bled formation (corresponding to site of separation of epithelium from lamina propria) is also detected in ethanol fed rats. It has been shown that alcohol inhibits protein synthesis after skeletal muscle contraction; therefore, alcohol could impair the repair on intestinal lining after alcohol‐induced gastric erosion.^60^

### 4.3 Tight junction integrity and ASH

A study on Caco‐2 cells (human colon adenocarcinoma cell line) demonstrated, in a cultured monolayer, that after exposure to ethanol, increased permeability was associated with reduced expression of Zonulin‐1 and Claudin‐1, two proteins of paramount importance to maintain tight junction integrity.^61^ Another study conducted on Occludin knock‐out mice showed increased ethanol damage in the IEB of these animals, suggesting a role also for this protein in maintaining a functional non‐leaky gut.^62^ During alcohol abuse, IEB alterations are thought to develop mainly because acetaldehyde toxicity. This actually determines loss of important intercellular proteins determining Adherens‐ and Tight‐junction damage and cytoskeleton rearrangement.^63^

**Figure 3:**
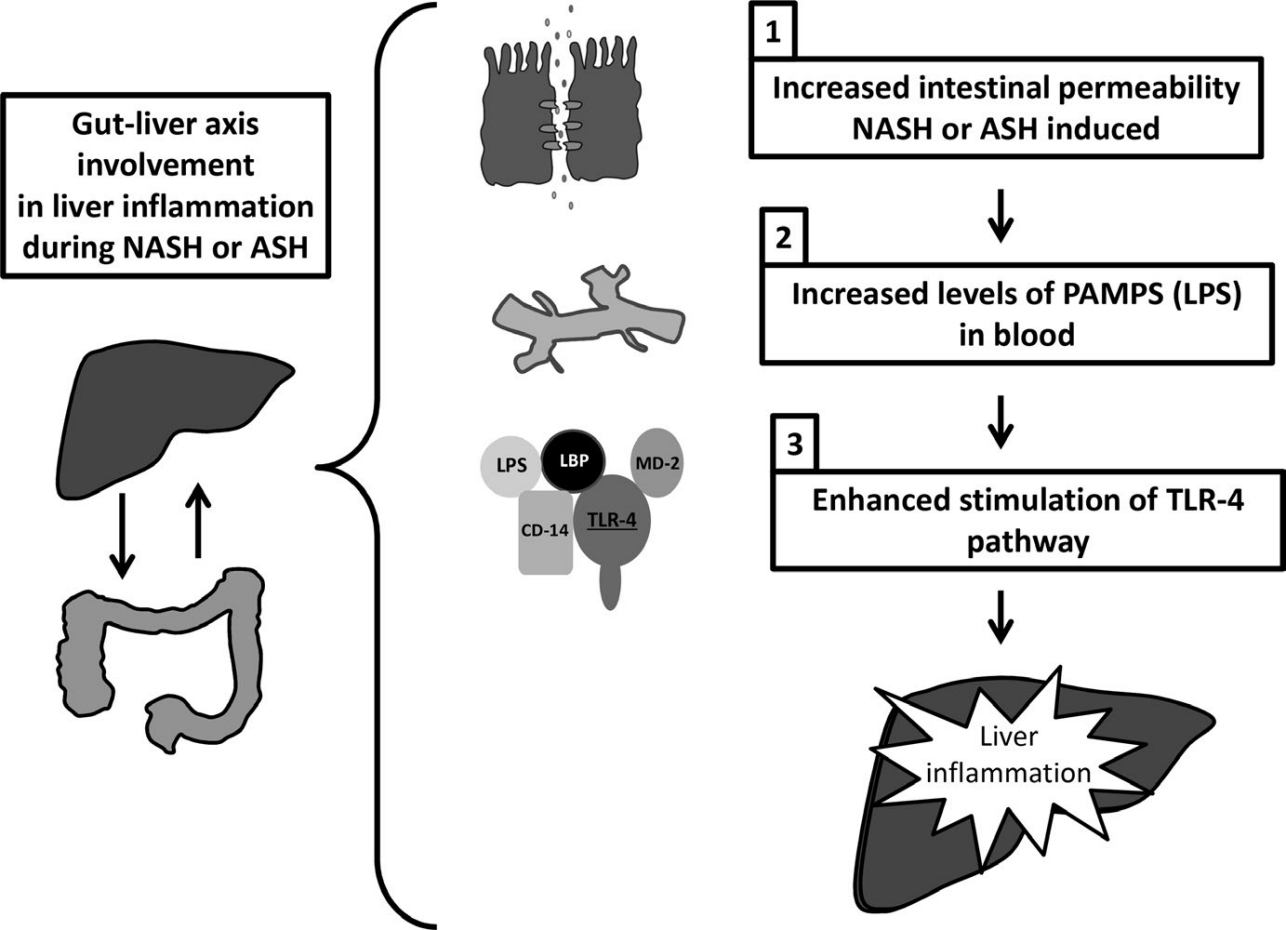
Schematic representation of gut‐liver axis role in NASH and ASH liver inflammation. (1) These diseases determine a condition of leaky gut as a consequence of the alteration of the normal bowel wall; (2) increased levels of PAMPS (LPS) are released in blood; (3) these in turn stimulate TLR4 pathway and liver inflammation. ASH, alcoholic steatohepatitis; LBP, LPS binding protein; LPS, lipopolysaccharide; NASH, non‐alcoholic steatohepatitis; PAMPs, pathogen‐associated molecular patterns; TLR4, Toll‐like receptor 4

From all of the above, even if a complete picture of the pathological mechanisms is still lacking, it is clear that gut‐liver axis derangement plays an important role in the development and progression of NASH and ASH. Future studies, targeting microbiota composition or intestinal permeability, may identify strategies to improve the outcome for these human liver diseases. The possible mechanism linking gut‐liver axis to liver injury in NASH and ASH is depicted in Figure 3.

## 5 OTHER RECENTLY ADVANCES POSSIBLY SUPPORTING INFLAMMATION IN NASH AND ASH

Table 1 comparatively reports the possible contributors to liver inflammation in NASH and ASH. Other factors have been recently suggested to influence inflammation in the course of NASH and ASH. Among these, adipokines and microRNAs are gaining interest for their possible role in the pathogenesis of these liver diseases. Recent findings are reported in the following paragraphs.

### 5.1 Adipokines

Visceral white adipose tissue (WAT), composed by cells deputed to handling of fats (mainly triglycerides and fatty acids), has an important metabolic interplay with the liver.^64^ Crosstalk between WAT and liver is mainly supported by an endocrine component of the first, determining the production of several peptide factors collectively named adipokines.^65^ WAT dysfunction is suggested in both NASH and ASH since an altered adipokines secretion has been described in these diseases.^66^, ^67^ While the secretion of more than 500 adipokines has been attributed to WAT, adiponectin and leptin are at present the most characterized with regard to their relationship with fatty liver. Adiponectin is a 247 amino acid peptide with reported anti‐inflammatory and anti‐atherogenic properties coupled with an insulin‐sensitizing activity. The effects are mainly mediated by interaction with the specific receptors ADIPOR 1 and 2 present in plasma membrane and in the human liver.^68^ Hepatic anti‐inflammatory effects of adiponectin have been linked to its binding to ADIPOR‐2 and inhibition of TLR‐4 through stimulation of 5′ AMP‐activated protein kinase (AMPK) and peroxisome proliferator–activated receptor‐α (PPAR alpha) pathways.^69^ Moreover, this adipokine is able to decrease tumour necrosis factor (TNF)‐alpha secretion in animal model of LPS‐induced liver injury^70^ and to increase the concentration of anti‐inflammatory cytokines (IL‐10 and IL‐1RA) in cultures of human monocytes, macrophages and dendritic cells.^71^ Adiponectin serum levels were examined in different studies on human NASH. A 2010 systematic review with meta‐analysis on this issue evidenced a statistically significant decrease in adiponectin serum levels in patients with NASH in comparison with healthy control or fatty liver subjects.^72^ On the basis of this finding, the authors proposed the decrease in adiponectin excretion as a possible contributor (hit) to the development and progression from fatty liver to NASH. In rodent experimental models of alcoholic liver injuries, similarly decreased adiponectin levels were reported^73^, ^74^; however, in humans a bimodal trend in adiponectin excretion has been described in regard to ethanol assumption, with increased levels in moderate drinkers^75^, ^76^ and a decrease in >90 g EtOH/d consumers.^77^, ^78^ Nevertheless, a clear relationship in humans, between high‐alcohol consumption and reduced adiponectin levels, has not been demonstrated so far and the possible presence of metabolic syndrome may be a confounding factor in this setting. In this perspective, a possible role of adiponectin in the pathogenesis of ASH is not likely.

Leptin is another important adipokine involved in lipolysis and fatty acid degradation; however, observation of leptin‐deficient rodents evidenced not only pathological obesity and fatty liver but also impairment in several physiologic functions (reproductive, angiogenetic, neuroendocrine and so on) including immunity. In fact, leptin is thought to contribute to immune function by stimulating inflammatory response and production of pro‐inflammatory cytokines.^79^ Possible mechanism in NASH involves a leptin/CD14‐mediated enhanced sensitivity to LPS as demonstrated in a murine model.^80^

A meta‐analysis on circulating levels of leptin in humans affected by fatty liver or NASH demonstrated increased levels of this hormone in the more severe form of the disease.^81^ In this perspective and with regard to NAFLD, a different role was proposed for leptin consisting in (a) prevention of fatty liver when excreted in adequate proportion (as suggested by leptin deficient mice) but (b) supporting inflammation and liver injury when its levels are increased, such as during NASH. With regard to ALD, leptin findings are more confusing. Different studies on levels of this hormone and alcohol intake gave conflicting results both in human and animal models.^77^ Even if a study on EtOH‐induced liver injury on male C57BL/6 mice demonstrated reduced damage after correction of leptin deficiency,^82^ at present a possible role for this hormone in the pathogenesis of human ASH remains uncertain.

Finally, while a competing mechanism maybe hypothesized between adiponectin and leptin in preventing or inducing liver damage in the course of NAFLD, data in regard of ASH are inconclusive and suggest a modest influence of these adipokines in alcohol‐related liver disease in human.

### 5.2 microRNAs

microRNAs (miRs), discovered in the early 90s, are small, non‐coding fragments of RNA acting at post‐transcriptional level and degrading or inhibiting the translation of a specific mRNA.^83^ Several miRs have been identified in the last decades, and they are thought to regulate several physiologic functions. On the other hand, environmental factors such as diet, exposure to drug or toxic may have a role in modulating miR expression. Recently, several studies focused on the possible role of miRs in NASH and ASH as reported in a recent review.^84^ miR‐122 is one of the most represented miRs in the human and rodent liver, with important functions in regard to development and homoeostatic maintenance of the graft.^85^ Data from miR‐122 knock‐out mice evidenced increased infiltration of fats, inflammation and carcinogenesis in the liver.^86^ In agreement with these observations, a study in humans with NASH (n = 25) evidenced a >50% decrease in liver miR‐122 expression in comparison with healthy control.^87^ Similar results were also observed in mice and humans with alcohol‐induced liver damage.^88^ In this latter study, in order to investigate the mechanism of miR‐122 decrease, the expression of grainyhead‐like transcription factor 2 (GRHL2) was evaluated. GRHL2 was previously found to decrease the expression of this miR in experimental setting of NASH evaluating differentiation of liver progenitor cells.^89^ Interestingly, an increased expression (nearly 20‐fold) of GRHL2 was observed in human liver, with alcoholic cirrhosis. Moreover, the GRHL2 increase was negatively correlated with miR‐122 expression in the same tissue.

On the basis of these results, the pathogenesis of ALD based on GRHL2‐mediated miR‐122 inhibition has been hypothesized. Other miRs such as miR‐34a have been evaluated in experimental models of NASH and ASH.^84^ miR‐34a levels were significantly up‐regulated in steatosis‐induced hepatocytes and in liver tissues of high‐fat diet‐fed mice as well as chronic ethanol feeding mice. Silencing miR‐34a led to an initially increased expression of PPARα, SIRT1 and PPARα’s downstream genes as well as central metabolic sensor AMPK. The miR‐34a inhibitor suppressed lipid accumulation and improved the degree of steatosis.^90^, ^91^ Indeed, some miRNAs seem to be altered in both diseases so that a common therapy for NASH and ASH (based on miR modulation) should be imagined in the future. The most relevant miRs altered in NASH and ASH are reported in Table 2. However, possible contribution of miRs altered expression in the pathogenesis of these liver affections remains to be unequivocally demonstrated. Moreover, these post‐transcriptional factors seem to act mainly as fine regulators of gene expression, sometimes lack specificity and a phenotypic clear effect, overall suggesting a partial contribution in these multifactorial diseases.

**Table 2:** More relevant microRNAs (miRs) involved in the pathogenesis of NASH and ASH with the corresponding function

miRs	NASH	ASH	Function
miR‐21	−	↑	↓apoptosis
miR‐34a	↑	↑	↑inflammation/necrosis
miR‐122	↓	↓	Regulation of liver physiology and lipid metabolism
miR‐155	↑	↑	Kupffer cell regulator
miR‐199	−	↑	↑inflammation
miR‐200a	−	↑	↑ apoptosis

Abbreviations: − unchanged; ↓ decrease; ASH, alcoholic steatohepatitis; miRs, microRNAs; NASH, non‐alcoholic steatohepatitis. Symbols: ↑ increase. Data on table were retrieved from the following References.^84^, ^87^

### 5.3 Extracellular vesicles

It has been demonstrated that a dynamic cell regulation may transpire as result of differentiated cell‐cell interaction via a extracellular vesicle‐based genetic information transfer. Hepatic cells may re‐direct the behaviour of differentiated cells by a horizontal transfer of mRNA or protein shuttled by EVs and conversely differentiated cells that may influence the development and progression of chronic liver injuries including NASH and ASH. EVs are derived from the endosomal membrane compartment after fusion with the plasma membrane and are shed from the cell surface of activated cells. Therefore, extracellular vesicles may be secreted by activated normal cells and play a role in cellular communication.^92^ EVs derived from liver cells may contribute to the cell‐fate decision and may represent one of the critical components that support expansion of various hepatic cells.^93^ Recent findings have also suggested that both NASH and ASH are characterized by an increase in circulating EVs. In order to characterize EV cargo, it was demonstrated that hepatocyte‐derived EVs released during lipotoxic fatty acids are enriched in the protein and microRNA that regulate the expression of PPAR‐gamma provoking an activation of stellate cells inducing fibrosis in the liver.^94^ Hepatocyte released EVs causing an increase in the percentage of F4/80+CD11b− (KCs) and TNF‐α, suggesting the link between pro‐inflammatory cytokines and hepatocyte intoxication during the process of ALD.^95^ The comprehensive analysis of liver cell‐derived EVs as the therapeutic agents in NASH and ASH will ultimately fill important gaps in our knowledge of the mechanisms of EVs in liver inflammation and gut‐liver axis and will address critical barriers to progress in the treatment of the patients with NASH and ASH.

## 6 CONCLUSIONS

We have summarized the translational mechanisms underlying pro‐inflammatory signalling and gut‐liver axis in non‐alcoholic and alcoholic steatohepatitis. NAFLD and ALD represent two important liver diseases in humans, with significantly associated mortality and morbidity. Clinical features are similar suggesting common mechanisms in the onset of these two conditions and possibly a common therapeutic strategy. However, deep examination of pro‐inflammatory processes in NASH and ASH reveals similarities but also important differences. The role of the gut‐liver axis is emerging as a major determinant of liver injury in NASH and ASH development and progression. Studies on this aspect will probably help to identify new and novel therapeutic strategies. Characterization of human microbiome remains an important factor to understand these and other liver and non‐liver diseases. For this reason, this remains the target of large ongoing, international projects. Another aspect that needs to be examined in deep in the future is that of gender difference in these diseases. Clinical data, for instance, support the view of a protective role of oestrogen against NAFLD since women in premenopausal state or in hormone replacement therapy are seldom affected by this disease.^96^ On the other hand, female sex, in humans, seems to increase the risk for toxic liver injury including the ethanol‐related one.^97^ Some immunological gender differences have been also identified in experimental studies. They may have a possible relationship with NAFLD and ASH damage. As an example, TLR4 is more expressed in normal condition and after stimulation in male human neutrophils.^98^ On the other hand, mouse female macrophages have an increased Myd88‐related response to LPS.^99^ Taken together, these findings recall our attention on the contribution of sex and sex hormones in the pathogenesis of these diseases as in other human affections. Research efforts on these aspects may be helpful to better understand the natural history of these conditions and to identify possible therapeutic targets.

## CONFLICTS OF INTEREST

The authors confirm that there are no conflicts of interest.

## AUTHOR CONTRIBUTIONS

TS, LB and FM performed the search and wrote the manuscript; TZ, HF, IL, GG, LK, SL and SG contributed to manuscript writing; and GA and FM supervised the work and wrote the manuscript. All authors read and approved the final manuscript.

## Data Availability

All the citations and data included in this manuscript are available upon request by contact with the corresponding author.

## References

[1] Wang YC , McPherson K , Marsh T , Gortmaker SL , Brown M . Health and economic burden of the projected obesity trends in the USA and the UK. Lancet. 2011;378:815‐825. 21872750 10.1016/S0140-6736(11)60814-3

[2] Chalasani N , Younossi Z , Lavine JE , et al. The diagnosis and management of nonalcoholic fatty liver disease: practice guidance from the American Association for the Study of Liver Diseases. Hepatology. 2018;67:328‐357. 28714183 10.1002/hep.29367

[3] Bellentani S , Scaglioni F , Marino M , Bedogni G . Epidemiology of non‐alcoholic fatty liver disease. Dig Dis. 2010;28:155‐161. 20460905 10.1159/000282080

[4] Williams CD , Stengel J , Asike MI , et al. Prevalence of nonalcoholic fatty liver disease and nonalcoholic steatohepatitis among a largely middle‐aged population utilizing ultrasound and liver biopsy: a prospective study. Gastroenterology. 2011;140:124‐131. 20858492 10.1053/j.gastro.2010.09.038

[5] Gao B , Bataller R . Alcoholic liver disease: pathogenesis and new therapeutic targets. Gastroenterology. 2011;141:1572‐1585. 21920463 10.1053/j.gastro.2011.09.002 PMC3214974

[6] Crabb DW , Im GY , Szabo G , Mellinger JL , Lucey MR . Diagnosis and treatment of alcohol‐related liver diseases: 2019 practice guidance from the American Association for the Study of Liver Diseases. Hepatology. 2020;71:306‐333. 31314133 10.1002/hep.30866

[7] Mann RE , Smart RG , Govoni R . The epidemiology of alcoholic liver disease. Alcohol Res Health. 2003;27:209‐219. 15535449 PMC6668879

[8] Lindenmeyer CC , McCullough AJ . The natural history of nonalcoholic fatty liver disease‐an evolving view. Clin Liver Dis. 2018;22:11‐21. 29128051 10.1016/j.cld.2017.08.003 PMC6130315

[9] Minuk GY , Sanders J , O'Brien M , Uhanova J . Estimated time from clinical presentation to the development of cirrhosis in non‐cirrhotic adult patients with non‐alcoholic fatty liver disease. Ann Hepatol. 2016;15:944‐945. 27740531 10.5604/16652681.1222117

[10] Kojima H , Sakurai S , Uemura M , et al. Difference and similarity between non‐alcoholic steatohepatitis and alcoholic liver disease. Alcohol Clin Exp Res. 2005;29:259S‐263S. 16385233 10.1097/01.alc.0000191776.37626.30

[11] Kawano Y , Cohen DE . Mechanisms of hepatic triglyceride accumulation in non‐alcoholic fatty liver disease. J Gastroenterol. 2013;48:434‐441. 23397118 10.1007/s00535-013-0758-5 PMC3633701

[12] Wang DQ , Portincasa P , Neuschwander‐Tetri BA . Steatosis in the liver. Compr Physiol. 2013;3:1493‐1532. 24265237 10.1002/cphy.c130001

[13] Javor ED , Ghany MG , Cochran EK , et al. Leptin reverses nonalcoholic steatohepatitis in patients with severe lipodystrophy. Hepatology. 2005;41:753‐760. 15791619 10.1002/hep.20672

[14] Donnelly KL , Smith CI , Schwarzenberg SJ , Jessurun J , Boldt MD , Parks EJ . Sources of fatty acids stored in liver and secreted via lipoproteins in patients with nonalcoholic fatty liver disease. J Clin Invest. 2005;115:1343‐1351. 15864352 10.1172/JCI23621 PMC1087172

[15] Morigny P , Houssier M , Mouisel E , Langin D . Adipocyte lipolysis and insulin resistance. Biochimie. 2016;125:259‐266. 26542285 10.1016/j.biochi.2015.10.024

[16] Steiner JL , Lang CH . Alcohol, adipose tissue and lipid dysregulation. Biomolecules. 2017;7(4):16.

[17] Teschke R . Alcoholic liver disease: alcohol metabolism, cascade of molecular mechanisms, cellular targets, and clinical aspects. Biomedicines. 2018;6:106. 10.3390/biomedicines6040106 PMC6316574 30424581

[18] Lieber CS . Alcoholic fatty liver: its pathogenesis and mechanism of progression to inflammation and fibrosis. Alcohol. 2004;34:9‐19. 15670660 10.1016/j.alcohol.2004.07.008

[19] Day CP , James OF . Steatohepatitis: a tale of two "hits"? Gastroenterology. 1998;114:842‐845. 9547102 10.1016/s0016-5085(98)70599-2

[20] Tilg H , Moschen AR . Evolution of inflammation in nonalcoholic fatty liver disease: the multiple parallel hits hypothesis. Hepatology. 2010;52:1836‐1846. 21038418 10.1002/hep.24001

[21] Mota M , Banini BA , Cazanave SC , Sanyal AJ . Molecular mechanisms of lipotoxicity and glucotoxicity in nonalcoholic fatty liver disease. Metabolism. 2016;65:1049‐1061. 26997538 10.1016/j.metabol.2016.02.014 PMC4931958

[22] Mendez‐Sanchez N , Cruz‐Ramon VC , Ramirez‐Perez OL , Hwang JP , Barranco‐Fragoso B , Cordova‐Gallardo J . New aspects of lipotoxicity in nonalcoholic steatohepatitis. Int J Mol Sci. 2018;19:2034. 10.3390/ijms19072034 PMC6073816 30011790

[23] Puri P , Baillie RA , Wiest MM , et al. A lipidomic analysis of nonalcoholic fatty liver disease. Hepatology. 2007;46:1081‐1090. 17654743 10.1002/hep.21763

[24] Caballero F , Fernandez A , De Lacy AM , Fernandez‐Checa JC , Caballeria J , Garcia‐Ruiz C . Enhanced free cholesterol, SREBP‐2 and StAR expression in human NASH. J Hepatol. 2009;50:789‐796. 19231010 10.1016/j.jhep.2008.12.016

[25] Brar G , Tsukamoto H . Alcoholic and non‐alcoholic steatohepatitis: global perspective and emerging science. J Gastroenterol. 2019;54:218‐225. 30643981 10.1007/s00535-018-01542-w PMC6394716

[26] Arguello G , Balboa E , Arrese M , Zanlungo S . Recent insights on the role of cholesterol in non‐alcoholic fatty liver disease. Biochim Biophys Acta. 2015;1852:1765‐1778. 26027904 10.1016/j.bbadis.2015.05.015

[27] Csak T , Ganz M , Pespisa J , Kodys K , Dolganiuc A , Szabo G . Fatty acid and endotoxin activate inflammasomes in mouse hepatocytes that release danger signals to stimulate immune cells. Hepatology. 2011;54:133‐144. 21488066 10.1002/hep.24341 PMC4158408

[28] Sunny NE , Parks EJ , Browning JD , Burgess SC . Excessive hepatic mitochondrial TCA cycle and gluconeogenesis in humans with nonalcoholic fatty liver disease. Cell Metab. 2011;14:804‐810. 22152305 10.1016/j.cmet.2011.11.004 PMC3658280

[29] Teodoro JS , Rolo AP , Duarte FV , Simoes AM , Palmeira CM . Differential alterations in mitochondrial function induced by a choline‐deficient diet: understanding fatty liver disease progression. Mitochondrion. 2008;8:367‐376. 18765303 10.1016/j.mito.2008.07.008

[30] Koliaki C , Szendroedi J , Kaul K , et al. Adaptation of hepatic mitochondrial function in humans with non‐alcoholic fatty liver is lost in steatohepatitis. Cell Metab. 2015;21:739‐746. 25955209 10.1016/j.cmet.2015.04.004

[31] Wei Y , Rector RS , Thyfault JP , Ibdah JA . Nonalcoholic fatty liver disease and mitochondrial dysfunction. World J Gastroenterol. 2008;14:193‐199. 18186554 10.3748/wjg.14.193 PMC2675113

[32] Paradies G , Paradies V , Ruggiero FM , Petrosillo G . Oxidative stress, cardiolipin and mitochondrial dysfunction in nonalcoholic fatty liver disease. World J Gastroenterol. 2014;20:14205‐14218. 25339807 10.3748/wjg.v20.i39.14205 PMC4202349

[33] Dudek J . Role of cardiolipin in mitochondrial signaling pathways. Front Cell Dev Biol. 2017;5:90. 29034233 10.3389/fcell.2017.00090 PMC5626828

[34] Ajith TA . Role of mitochondria and mitochondria‐targeted agents in non‐alcoholic fatty liver disease. Clin Exp Pharmacol Physiol. 2018;45:413‐421. 29112771 10.1111/1440-1681.12886

[35] Sanyal AJ , Campbell‐Sargent C , Mirshahi F , et al. Nonalcoholic steatohepatitis: association of insulin resistance and mitochondrial abnormalities. Gastroenterology. 2001;120:1183‐1192. 11266382 10.1053/gast.2001.23256

[36] Sharifnia T , Antoun J , Verriere TG , et al. Hepatic TLR4 signaling in obese NAFLD. Am J Physiol Gastrointest Liver Physiol. 2015;309:G270‐G278. 26113297 10.1152/ajpgi.00304.2014 PMC4537925

[37] Cai J , Zhang XJ , Li H . Role of innate immune signaling in non‐alcoholic fatty liver disease. Trends Endocrinol Metab. 2018;29:712‐722. 30131212 10.1016/j.tem.2018.08.003

[38] Rivera CA , Adegboyega P , van Rooijen N , Tagalicud A , Allman M , Wallace M . Toll‐like receptor‐4 signaling and Kupffer cells play pivotal roles in the pathogenesis of non‐alcoholic steatohepatitis. J Hepatol. 2007;47:571‐579. 17644211 10.1016/j.jhep.2007.04.019 PMC2094119

[39] Kiziltas S , Ata P , Colak Y , et al. TLR4 gene polymorphism in patients with nonalcoholic fatty liver disease in comparison to healthy controls. Metab Syndr Relat Disord. 2014;12:165‐170. 24443993 10.1089/met.2013.0120

[40] Wan Y , Garner J , Wu N , et al. Role of stem cells during diabetic liver injury. J Cell Mol Med. 2016;20:195‐203. 26645107 10.1111/jcmm.12723 PMC4727564

[41] Wree A , McGeough MD , Pena CA , et al. NLRP3 inflammasome activation is required for fibrosis development in NAFLD. J Mol Med (Berl). 2014;92:1069‐1082. 24861026 10.1007/s00109-014-1170-1 PMC4349416

[42] Spruss A , Kanuri G , Wagnerberger S , Haub S , Bischoff SC , Bergheim I . Toll‐like receptor 4 is involved in the development of fructose‐induced hepatic steatosis in mice. Hepatology. 2009;50:1094‐1104. 19637282 10.1002/hep.23122

[43] Hritz I , Mandrekar P , Velayudham A , et al. The critical role of toll‐like receptor (TLR) 4 in alcoholic liver disease is independent of the common TLR adapter MyD88. Hepatology. 2008;48:1224‐1231. 18792393 10.1002/hep.22470 PMC7137387

[44] Hoyt LR , Ather JL , Randall MJ , et al. Ethanol and other short‐chain alcohols inhibit NLRP3 inflammasome activation through protein tyrosine phosphatase stimulation. J Immunol. 2016;197:1322‐1334. 27421477 10.4049/jimmunol.1600406 PMC4975963

[45] Nurmi K , Virkanen J , Rajamaki K , Niemi K , Kovanen PT , Eklund KK . Ethanol inhibits activation of NLRP3 and AIM2 inflammasomes in human macrophages–a novel anti‐inflammatory action of alcohol. PLoS ONE. 2013;8:e78537. 24244322 10.1371/journal.pone.0078537 PMC3823849

[46] Lippai D , Bala S , Petrasek J , et al. Alcohol‐induced IL‐1beta in the brain is mediated by NLRP3/ASC inflammasome activation that amplifies neuroinflammation. J Leukoc Biol. 2013;94:171‐182. 23625200 10.1189/jlb.1212659 PMC3685015

[47] Ursell LK , Metcalf JL , Parfrey LW , Knight R . Defining the human microbiome. Nutr Rev. 2012;70(Suppl 1):S38‐44. 22861806 10.1111/j.1753-4887.2012.00493.x PMC3426293

[48] Peterson J , Garges S , Giovanni M , et al. The NIH human microbiome project. Genome Res. 2009;19:2317‐2323. 19819907 10.1101/gr.096651.109 PMC2792171

[49] Balmer ML , Slack E , de Gottardi A , et al. The liver may act as a firewall mediating mutualism between the host and its gut commensal microbiota. Sci Transl Med. 2014;6:237ra66. 10.1126/scitranslmed.3008618 24848256

[50] Cani PD , Amar J , Iglesias MA , et al. Metabolic endotoxemia initiates obesity and insulin resistance. Diabetes. 2007;56:1761‐1772. 17456850 10.2337/db06-1491

[51] Hu X , Wang T , Liang S , Li W , Wu X , Jin F . Antibiotic‐induced imbalances in gut microbiota aggravates cholesterol accumulation and liver injuries in rats fed a high‐cholesterol diet. Appl Microbiol Biotechnol. 2015;99:9111‐9122. 26129950 10.1007/s00253-015-6753-4

[52] Ohtani N , Kawada N . Role of the gut‐liver axis in liver inflammation, fibrosis, and cancer: a special focus on the gut microbiota relationship. Hepatol Commun. 2019;3:456‐470. 30976737 10.1002/hep4.1331 PMC6442695

[53] Brandl K , Kumar V , Eckmann L . Gut‐liver axis at the frontier of host‐microbial interactions. Am J Physiol Gastrointest Liver Physiol. 2017;312:G413‐G419. 28232456 10.1152/ajpgi.00361.2016 PMC5451561

[54] Schwabe RF , Seki E , Brenner DA . Toll‐like receptor signaling in the liver. Gastroenterology. 2006;130:1886‐1900. 16697751 10.1053/j.gastro.2006.01.038

[55] Poltorak A , He X , Smirnova I , et al. Defective LPS signaling in C3H/HeJ and C57BL/10ScCr mice: mutations in Tlr4 gene. Science. 1998;282:2085‐2088. 9851930 10.1126/science.282.5396.2085

[56] Soares JB , Pimentel‐Nunes P , Roncon‐Albuquerque R , Leite‐Moreira A . The role of lipopolysaccharide/toll‐like receptor 4 signaling in chronic liver diseases. Hepatol Int. 2010;4:659‐672. 21286336 10.1007/s12072-010-9219-x PMC2994611

[57] Liangpunsakul S , Toh E , Ross RA , et al. Quantity of alcohol drinking positively correlates with serum levels of endotoxin and markers of monocyte activation. Sci Rep. 2017;7:4462. 28667254 10.1038/s41598-017-04669-7 PMC5493657

[58] Wigg AJ , Roberts‐Thomson IC , Dymock RB , McCarthy PJ , Grose RH , Cummins AG . The role of small intestinal bacterial overgrowth, intestinal permeability, endotoxaemia, and tumour necrosis factor alpha in the pathogenesis of non‐alcoholic steatohepatitis. Gut. 2001;48:206‐211. 11156641 10.1136/gut.48.2.206 PMC1728215

[59] Mouries J , Brescia P , Silvestri A , et al. Microbiota‐driven gut vascular barrier disruption is a prerequisite for non‐alcoholic steatohepatitis development. J Hepatol. 2019;71:1216‐1228. 31419514 10.1016/j.jhep.2019.08.005 PMC6880766

[60] Pruznak AM , Nystrom J , Lang CH . Direct central nervous system effect of alcohol alters synthesis and degradation of skeletal muscle protein. Alcohol Alcohol. 2013;48:138‐145. 23079499 10.1093/alcalc/ags113 PMC3571205

[61] Wang Y , Tong J , Chang B , Wang B , Zhang D , Wang B . Effects of alcohol on intestinal epithelial barrier permeability and expression of tight junction‐associated proteins. Mol Med Rep. 2014;9:2352‐2356. 24718485 10.3892/mmr.2014.2126

[62] Mir H , Meena AS , Chaudhry KK , et al. Occludin deficiency promotes ethanol‐induced disruption of colonic epithelial junctions, gut barrier dysfunction and liver damage in mice. Biochim Biophys Acta. 2016;1860:765‐774. 26721332 10.1016/j.bbagen.2015.12.013 PMC4776745

[63] Rao R . Endotoxemia and gut barrier dysfunction in alcoholic liver disease. Hepatology. 2009;50:638‐644. 19575462 10.1002/hep.23009 PMC6209509

[64] Scheja L , Heeren J . Metabolic interplay between white, beige, brown adipocytes and the liver. J Hepatol. 2016;64:1176‐1186. 26829204 10.1016/j.jhep.2016.01.025

[65] Ouchi N , Parker JL , Lugus JJ , Walsh K . Adipokines in inflammation and metabolic disease. Nat Rev Immunol. 2011;11:85‐97. 21252989 10.1038/nri2921 PMC3518031

[66] Pravdova E , Fickova M . Alcohol intake modulates hormonal activity of adipose tissue. Endocr Regul. 2006;40:91‐104. 17100551

[67] van der Poorten D , Milner KL , Hui J , et al. Visceral fat: a key mediator of steatohepatitis in metabolic liver disease. Hepatology. 2008;48:449‐457. 18627003 10.1002/hep.22350

[68] Felder TK , Hahne P , Soyal SM , et al. Hepatic adiponectin receptors (ADIPOR) 1 and 2 mRNA and their relation to insulin resistance in obese humans. Int J Obesity. 2005;2010(34):846‐851. 10.1038/ijo.2010.7 20125105

[69] Polyzos SA , Kountouras J , Zavos C , Tsiaousi E . The role of adiponectin in the pathogenesis and treatment of non‐alcoholic fatty liver disease. Diabetes Obes Metab. 2010;12:365‐383. 20415685 10.1111/j.1463-1326.2009.01176.x

[70] Masaki T , Chiba S , Tatsukawa H , et al. Adiponectin protects LPS‐induced liver injury through modulation of TNF‐alpha in KK‐Ay obese mice. Hepatology. 2004;40:177‐184. 15239101 10.1002/hep.20282

[71] Wolf AM , Wolf D , Rumpold H , Enrich B , Tilg H . Adiponectin induces the anti‐inflammatory cytokines IL‐10 and IL‐1RA in human leukocytes. Biochem Biophys Res Commun. 2004;323:630‐635. 15369797 10.1016/j.bbrc.2004.08.145

[72] Polyzos SA , Toulis KA , Goulis DG , Zavos C , Kountouras J . Serum total adiponectin in nonalcoholic fatty liver disease: a systematic review and meta‐analysis. Metabolism. 2011;60:313‐326. 21040935 10.1016/j.metabol.2010.09.003

[73] Chen X , Sebastian BM , Tang H , et al. Taurine supplementation prevents ethanol‐induced decrease in serum adiponectin and reduces hepatic steatosis in rats. Hepatology. 2009;49:1554‐1562. 19296466 10.1002/hep.22811 PMC2677130

[74] Tian C , Jin X , Ye X , et al. Long term intake of 0.1% ethanol decreases serum adiponectin by suppressing PPARgamma expression via p38 MAPK pathway. Food Chem Toxicol. 2014;65:329‐334. 24412557 10.1016/j.fct.2014.01.007

[75] Bell S , Britton A . The role of alcohol consumption in regulating circulating levels of adiponectin: a prospective cohort study. J Clin Endocrinol Metab. 2015;100:2763‐2768. 26000546 10.1210/jc.2015-1845 PMC4490299

[76] Sierksma A , Patel H , Ouchi N , et al. Effect of moderate alcohol consumption on adiponectin, tumor necrosis factor‐alpha, and insulin sensitivity. Diabetes Care. 2004;27:184‐189. 14693987 10.2337/diacare.27.1.184

[77] Hansen KB , Ingerslev M , Larsen JF , Pedersen GT . [Clomiphene in the treatment of patients with anovular sterility]. Ugeskr Laeger. 1969;131:2253‐2256. 5372728

[78] Jung SK , Kim MK , Shin J , Choi BY . A cross‐sectional analysis of the relationship between daily alcohol consumption and serum adiponectin levels among adults aged 40 years or more in a rural area of Korea. Eur J Clin Nutr. 2013;67:841‐847. 23612511 10.1038/ejcn.2013.74

[79] Procaccini C , Galgani M , De Rosa V , et al. Leptin: the prototypic adipocytokine and its role in NAFLD. Curr Pharm Des. 2010;16:1902‐1912. 20370676 10.2174/138161210791208884

[80] Imajo K , Fujita K , Yoneda M , et al. Hyperresponsivity to low‐dose endotoxin during progression to nonalcoholic steatohepatitis is regulated by leptin‐mediated signaling. Cell Metab. 2012;16:44‐54. 22768838 10.1016/j.cmet.2012.05.012

[81] Polyzos SA , Aronis KN , Kountouras J , Raptis DD , Vasiloglou MF , Mantzoros CS . Circulating leptin in non‐alcoholic fatty liver disease: a systematic review and meta‐analysis. Diabetologia. 2016;59:30‐43. 26407715 10.1007/s00125-015-3769-3

[82] Tan X , Sun X , Li Q , et al. Leptin deficiency contributes to the pathogenesis of alcoholic fatty liver disease in mice. Am J Pathol. 2012;181:1279‐1286. 22841822 10.1016/j.ajpath.2012.06.013 PMC3463622

[83] Ambros V . The functions of animal microRNAs. Nature. 2004;431:350‐355. 15372042 10.1038/nature02871

[84] Torres JL , Novo‐Veleiro I , Manzanedo L , et al. Role of microRNAs in alcohol‐induced liver disorders and non‐alcoholic fatty liver disease. World J Gastroenterol. 2018;24:4104‐4118. 30271077 10.3748/wjg.v24.i36.4104 PMC6158486

[85] Bandiera S , Pfeffer S , Baumert TF , Zeisel MB . miR‐122–a key factor and therapeutic target in liver disease. J Hepatol. 2015;62:448‐457. 25308172 10.1016/j.jhep.2014.10.004

[86] Hsu SH , Wang B , Kota J , et al. Essential metabolic, anti‐inflammatory, and anti‐tumorigenic functions of miR‐122 in liver. J Clin Invest. 2012;122:2871‐2883. 22820288 10.1172/JCI63539 PMC3408748

[87] Cheung O , Puri P , Eicken C , et al. Nonalcoholic steatohepatitis is associated with altered hepatic MicroRNA expression. Hepatology. 2008;48:1810‐1820. 19030170 10.1002/hep.22569 PMC2717729

[88] Satishchandran A , Ambade A , Rao S , et al. MicroRNA 122, regulated by GRLH2, protects livers of mice and patients from ethanol‐induced liver disease. Gastroenterology. 2018;154:238 ‐ 252.e7. 28987423 10.1053/j.gastro.2017.09.022 PMC5742049

[89] Tanimizu N , Kobayashi S , Ichinohe N , Mitaka T . Downregulation of miR122 by grainyhead‐like 2 restricts the hepatocytic differentiation potential of adult liver progenitor cells. Development. 2014;141:4448‐4456. 25406394 10.1242/dev.113654

[90] Ding J , Li M , Wan X , et al. Effect of miR‐34a in regulating steatosis by targeting PPARalpha expression in nonalcoholic fatty liver disease. Sci Rep. 2015;5:13729. 26330104 10.1038/srep13729 PMC4557122

[91] Meng F , Glaser SS , Francis H , et al. Epigenetic regulation of miR‐34a expression in alcoholic liver injury. Am J Pathol. 2012;181:804‐817. 22841474 10.1016/j.ajpath.2012.06.010 PMC3432440

[92] Ratajczak J , Wysoczynski M , Hayek F , Janowska‐Wieczorek A , Ratajczak MZ . Membrane‐derived microvesicles: important and underappreciated mediators of cell‐to‐cell communication. Leukemia. 2006;20:1487‐1495. 16791265 10.1038/sj.leu.2404296

[93] Ratajczak J , Miekus K , Kucia M , et al. Embryonic stem cell‐derived microvesicles reprogram hematopoietic progenitors: evidence for horizontal transfer of mRNA and protein delivery. Leukemia. 2006;20:847‐856. 16453000 10.1038/sj.leu.2404132

[94] Moran L , Cubero FJ . Extracellular vesicles in liver disease and beyond. World J Gastroenterol. 2018;24:4519‐4526. 30386101 10.3748/wjg.v24.i40.4519 PMC6209575

[95] Saha B , Momen‐Heravi F , Furi I , et al. Extracellular vesicles from mice with alcoholic liver disease carry a distinct protein cargo and induce macrophage activation through heat shock protein 90. Hepatology. 2018;67:1986‐2000. 29251792 10.1002/hep.29732 PMC5906190

[96] Lonardo A , Nascimbeni F , Ballestri S , et al. Sex differences in nonalcoholic fatty liver disease: state of the art and identification of research gaps. Hepatology. 2019;70:1457‐1469. 30924946 10.1002/hep.30626 PMC6766425

[97] Guy J , Peters MG . Liver disease in women: the influence of gender on epidemiology, natural history, and patient outcomes. Gastroenterol Hepatol (N Y). 2013;9:633‐639. 24764777 PMC3992057

[98] Aomatsu M , Kato T , Kasahara E , Kitagawa S . Gender difference in tumor necrosis factor‐alpha production in human neutrophils stimulated by lipopolysaccharide and interferon‐gamma. Biochem Biophys Res Commun. 2013;441:220‐225. 24140406 10.1016/j.bbrc.2013.10.042

[99] Klein SL , Marriott I , Fish EN . Sex‐based differences in immune function and responses to vaccination. Trans R Soc Trop Med Hyg. 2015;109:9‐15. 25573105 10.1093/trstmh/tru167 PMC4447843

